# Child marriage and relationship quality in Ethiopia

**DOI:** 10.1080/13691058.2018.1520919

**Published:** 2018-11-09

**Authors:** Neetu A. John, Jeffrey Edmeades, Lydia Murithi, Iman Barre

**Affiliations:** International Center for Research on Women, Washington DC, DC, USA

**Keywords:** Child marriage, relationship quality, Ethiopia, Africa

## Abstract

Child marriage is prevalent in Africa, with almost 40% of girls being married before age 18. Although child marriage is linked to a range of adverse outcomes, including intimate partner violence, little is known about the quality of these marriages in terms of the levels of communication, trust, equality, intimacy, conflict, marital satisfaction or happiness. We used both quantitative and qualitative data to examine how exact age at first marriage influenced multiple domains of relationship quality in Ethiopia. Our analysis was based on household survey data from 3396 currently married or recently divorced women aged 18–45, 32 in-depth interviews and 8 participatory focus groups in two regions. The regression results show a strong negative effect of marriage at or before age 12 on relationship quality across multiple domains. The qualitative data suggest a more pervasive effect on marital quality, with the lack of ability to choose whom they married and reduced agency emerging as particularly important factors influencing marital quality. This relationship may be direct or indirect, potentially mediated by factors such as intimate partner violence. Interventions intending to mitigate the effects of child marriage should include components that aim to improve the quality of spousal relationships, particularly in terms of communication and negotiation skills.

## Résumé

Le mariage d’enfants est largement répandu en Afrique, avec presque 40 % des filles qui se retrouvent mariées avant l’âge de 18 ans. Bien que les mariages d’enfants soient associés à une série de conséquences négatives incluant la violence entre partenaires intimes, les connaissances sont limitées sur leur qualité, en termes de niveaux de communication, de confiance, d’égalité, d’intimité, de conflits, de satisfaction conjugale ou de bonheur conjugal. Nous avons utilisé à la fois des données quantitatives et qualitatives pour examiner comment l’âge exact au premier mariage influençait plusieurs domaines de la qualité des relations en Éthiopie. Ces données provenaient d’une enquête sur les ménages : 3396 femmes actuellement mariées ou récemment divorcées, âgées de 18 à 45 ans, 32 entretiens en profondeur et huit groupes de discussion thématique participatifs ayant été conduits dans deux régions. Les résultats de la régression montrent un impact négatif puissant du mariage à l’âge de 12 ans (ou avant) sur la qualité de la relation, transversalement à des domaines multiples. Les données qualitatives suggèrent un effet plus généralisé sur la qualité du mariage, l’absence de capacité à choisir son mari et la diminution de la capacité à agir émergeant comme des facteurs d’influence sur la qualité du mariage particulièrement importants. Cette relation peut être directe ou indirecte, potentiellement tempérée par des facteurs tes que la violence entre partenaires intimes. Les interventions qui visent à pondérer les effets du mariage d’enfants doivent inclure des composantes qui ciblent l’amélioration de la qualité des relations conjugales, en particulier en termes de communication et de compétences pour négocier.

## Resumen

El matrimonio infantil es prevalente en África y casi un 40% de las niñas se casan antes de llegar a los 18 años. Aunque se vincula el matrimonio infantil con una serie de consecuencias perjudiciales, incluyendo la violencia de pareja, poco se sabe sobre la calidad de estos matrimonios en lo que respecta a los niveles de comunicación, confianza, igualdad, intimidad, conflicto, satisfacción matrimonial o felicidad. Mediante datos cuantitativos y cualitativos llevamos a cabo un análisis de cómo afecta la edad exacta en el primer matrimonio en los múltiples dominios de la calidad en las relaciones en Etiopía. Esto se basó en los datos de una encuesta de hogares en la que participaron 3396 mujeres recién casadas o que se acababan de divorciar con edades comprendidas entre los 18 y 45 años, 32 entrevistas exhaustivas y 8 charlas en grupo en dos regiones. Los resultados de regresión muestran un efecto claramente negativo del matrimonio en niñas casadas antes de los 12 años en la calidad de las relaciones en muchos dominios. Los datos cualitativos indican un efecto más dominante en la calidad del matrimonio, con la falta de capacidad para elegir con quién se casan y una reducida capacidad de acción surgiendo como factores especialmente importantes que influían en la calidad del matrimonio. Esta relación puede ser directa o indirecta, mediada probablemente por factores como la violencia de pareja. En los programas destinados a mitigar los efectos del matrimonio infantil se deberían incluir componentes que tengan como finalidad mejorar la calidad de las relaciones entre los esposos, sobre todo en lo que respecta a las habilidades de comunicación y negociación.

## Introduction

Child marriage, defined as marriage before the age of 18 years, continues to be prevalent in Africa, where almost 40% of girls marry before reaching this age (UNICEF 2014). Marriage triggers unique changes in the lives of girls and requires abrupt transitioning into adult roles and responsibilities before they may be ready to tackle these responsibilities. Research has consistently documented the adverse economic, social, demographic and health consequences of child marriage for child brides, their families and communities (for a review see Parsons et al. 2015). Evidence also suggests that girls who are most likely to marry early come from poor households, live in rural areas and have limited opportunities for schooling and labour force participation (UNICEF 2014; Parsons et al. 2015; Erulkar 2013). Due to these factors, these young girls tend to enter marriage with limited assets and resources, ultimately leaving them poorly equipped to navigate their new adult marital roles and exercise choice and agency in their lives and relationships (Choi and Ha 2011; Fleming, White and Catalano 2010). While it is clear that factors associated with child marriage make the transition to marriage and adult roles harder for child brides, not much is known about the nature and conditions of these marriages, despite there being an estimated 700 million child brides worldwide (UNICEF 2014), and the potential importance of this knowledge for efforts aimed at mitigating some of the negative impacts associated with child marriage.

Current evidence suggests that in many contexts the types of marriages child brides enter into place them in a uniquely vulnerable position. Child marriages are often arranged by the parents and/or the family of the bride and tend to be polygamous marital arrangements. Child brides are also more likely to have larger age differences with their husbands than women who marry after the age of 18 (Erulkar 2013; Jensen and Thornton 2003). Each of these outcomes can further intensify power imbalances within their marriages and limit their autonomy and decision-making ability (Erulkar 2013; Jensen and Thornton 2003). Not surprisingly, evidence suggests that women who marry early are often at an increased risk of experiencing intimate partner violence (IPV). A study using standardised data from Demographic and Health Surveys (DHS) in 34 countries found that young women (age 20–24) who married as children were at increased risk of past year physical and/or sexual IPV as compared to women who married as adults (Kidman 2016). More recently, a multi-country analysis based on demographic and health surveys as well as data collected as part of the current study suggests that very early marriage (at or under 15 years of age) is positively associated with increased risk of IPV and diminished decision-making ability (Wodon et al. 2017). Although we have some understanding of how child marriages perpetuate gendered power differences between spouses and their adverse effects, we have limited understanding of the ‘quality’ of these relationships, such as the level of emotional engagement they involve, despite the potential influence this may have on each of the behaviours and outcomes described above as well as in supporting healthy behaviours within couples.

In the West, there is a growing body of literature on the benefits of higher-quality marriages, ranging from improved overall health and psychological well-being to reduced mortality (Robles 2014; Strine et al. 2008; Choi and Ha 2011). Studies also suggest that individuals in higher-quality marriages are less likely to engage in unhealthy behaviours such as substance abuse and excessive drinking and are more likely to practise healthy behaviours such as healthy eating and the consistent use of contraception (Fleming, White and Catalano 2010; Whisman, Uebelacker and Bruce 2006; Manlove et al. 2011). While evidence from sub-Saharan Africa is sparse, there is a small body of work emerging that highlights the potential of this understudied topic for the region. For example, recent studies in Ethiopia and South Africa point to the relevance of focusing on relationship quality (John et al. 2016; Conroy et al. 2016; Conroy et al. 2017). Similarly, while a study from Ethiopia found that partner relationship quality influenced each partner’s health and well-being, another study from Ghana found a positive association between relationship quality and contraceptive use (John et al. 2017; Cox et al. 2013).

Understanding how child marriage influences relationship quality will not only shed light on this neglected subject in sub-Saharan Africa but can also potentially help illuminate the mechanisms through which child marriage adversely impacts the health and well-being of child brides and their families. This knowledge can prove useful for designing programmes and interventions to mitigate some of the negative outcomes associated with child marriage. Given this evidence gap, we used data from a large-scale population-based survey in Ethiopia, covering nine regions and one town administrative area of the country, which surveyed 4129 ever-married women aged 18–45 years to examine how child marriage influences relationship quality. These data were supplemented by qualitative data designed to explore the consequences of child marriage in an urban and a rural site in Oromia and Amhara regions, respectively.

Measures of relationship quality and its related constructs have been the subject of much analysis in family research in North America. Although debates continue, in the Western literature relationship quality is typically defined as a multi-dimensional construct that measures both objective characteristics of marital relationships, such as levels of communication, intimacy, trust, equality and conflict, and subjective aspects like marital satisfaction or happiness (Lewis and Spanier 1979; Glenn 1989). We adopted this approach to define relationship quality. We also used exact age at marriage, rather than the more typical groupings of those married before ages 15 and 18, to allow for a more nuanced understanding of the gradient of risk at different ages, given the rapid physical and sexual changes and brain development that characterise adolescence.

### Early marriage in Ethiopia

While the proportion of girls marrying as children is declining slowly at the global level, there has been faster progress in Ethiopia in recent decades. Nevertheless, over half of Ethiopian women (58%) were married before the age of 18 in 2016 (Central Statistical Agency (CSA) [Ethiopia] and ICF 2016. Moreover, as many as 11% of married women are in polygynous unions, and the share of women who marry early tends to be higher in polygamous as compared to monogamous households (CSA [Ethiopia] and ICF 2016). Although there is variation in marriage customs across religious and ethnic groups, marriages are often traditionally arranged by families, with very brief engagement periods (Tilson and Larsen 2000). The bride joins the groom’s home until the couple establishes their own household (Ezra 2003). Divorce is prevalent and, as per one estimate, as many as 45% of first marriages end in divorce within the first 30 years (Tilson and Larsen 2000).

## Methods

### Data

Data for this study were derived from a multi-country study aimed at estimating the economic costs of child marriage. At the global level, ethical review was provided by the Institutional Review Board at the International Center for Research on Women, while in Ethiopia the review was provided by the National Research Ethics Review Committee. The study surveyed ever-married women in the age range of 18–45 years. In Ethiopia, a total of 4149 women were interviewed in nine regions and one town administrative unit of the country. A three-stage stratified sampling approach was used to develop the sample. First, within each region, districts were selected by probability proportional to size, followed by random selection of counties and villages. A household census was then conducted within each village, followed by random selection of 25 households. If a woman did not consent to participate, the field team selected another eligible woman from the same household or an adjacent household. In cases where a family head had multiple wives, only one randomly selected wife was interviewed. The women’s questionnaire collected detailed information on the woman’s demographic background, health history, relationship with her husband and experience with IPV. In addition, the household questionnaire collected information on household wealth, along with demographic and health information of the household members.

The quantitative data were complemented by detailed qualitative data, collected via in-depth interviews (IDIs) and participatory focus group discussions (FGDs) in an urban and a rural site in Oromia and Amhara regions. While rural areas of Oromia have some of the highest prevalences of child marriage, urban areas of Amhara have some of the lowest prevalences in the country. The sample was equally split between the two study sites. In addition, to align with the quantitative data collection, only women aged 18–45 were included in the IDIs. Furthermore, to capture a range of marital experiences, only women who had been married for at least five years at the time of interview were included. Only parents (fathers and mothers) of daughters in the age range of 8–17 years were eligible to participate in the FGDs. As is commonly practised in qualitative research, purposive sampling was used to enrol participants. Potential participants were identified by community facilitators and screened by the research team. The team also used snowballing sampling methods, in which existing study subjects recruit from among their acquaintances, with the support from the facilitators, for further recruitment. A total of 32 IDIs (16 in each region) and 8 FGDs (2 with fathers and 2 with mothers in each region) were completed. Of the 32 IDIs completed, half were with women aged 18–24 and the other half aged 25–45. These data were used to further explore topics, including relationship quality, where the quantitative evidence for a causal link with child marriage was particularly lacking or where survey data were unlikely to provide sufficient nuance.

### Quantitative measures

#### Key dependent variables

*Relationship quality*: Measures of relationship quality included three scales that have previously been used in the African context that measure domains of trust, intimacy and equity in a relationship (John et al. 2017; Conroy et al. 2016; John, Seme and Tsui 2016). In addition, we also included a measure to assess general spousal communication.

The trust scale used was an eight-item scale developed by Larzelere and Huston in 1980, itself an adaptation of the Dyadic Trust Scale (Larzelere and Huston 1980). They define trust ‘as a belief by a person in the integrity of another individual’. The scale was conceptualised as a one-dimensional construct and has been shown to be associated with love and intimacy. Respondents were asked, on a scale of one to seven, to indicate how much they agree with each statement, with higher scores associated with greater trust perceived by the respondent in her/his relationship. The positively worded items were reverse-coded.

Intimacy and equality were both measured using sub-scales of the Relationship Values Scale developed by Kurdek (1996). The intimacy scale consists of six statements, and respondents were asked, on a scale of one to nine, to indicate how much they agree with each statement, with higher scores indicating more intimacy. The equality scale consists of eight items and is designed to capture the extent to which power and responsibility are shared between partners in the relationship. Respondents were asked, on a scale from one to nine, to indicate how much they agreed with each statement, with higher scores indicating greater equality between partners.

To assess general spousal communication, respondents were asked if they and their spouse communicated with each other about how their day went, their fears and worries, hopes for the future, things that happen in the community, birth spacing, schooling and health needs of children. The reliability and validity of the scales were assessed using Cronbach’s alpha and exploratory factor analysis (see Appendix 1 for included items and loadings). The predicted scores obtained following factor analysis were normalised to range from 0 to 100, where a higher score indicates better relationship quality.

### Key independent variables

*Child marriage*: To examine the gradient of risk based on age at marriage, a series of dummy variables were created to represent women who were married at 12 years or earlier, at 13 years, at 14 years, at 15 years, at 16 years and at 17 years.

*Intimate partner violence (IPV)*: The IPV measures were drawn from the 2004 World Health Organization’s (WHO) Multi-Country Study on Women’s Health and Domestic Violence against Women (García-Moreno 2005). The survey included measures of physical, sexual and emotional violence. The respondents were asked to report on both IPV experienced in the 12 months preceding the survey and prior to that point. For this analysis, we only combined measures of physical and sexual violence, which were created using principal components analysis (PCA), with the predicted scores normalised to range from 0 to 100, where a higher score indicated more violence.

*Spousal age difference*: To generate this variable, the wife’s age was subtracted from her partner’s age.

*Polygamous marriage*: A dummy variable was created to differentiate between polygamous and monogamous marriage.

*Community-level norms*: Measures of child marriage and relationship quality were aggregated from individual responses, excluding the index respondent’s response.

In addition, a range of standard socio-demographic, household and couple-level variables known to influence relationship quality and child marriage were included in the analysis such as the respondents’ age, education, number of children, household wealth and religion.

### Statistical analysis

We conducted a series of multivariate linear regression analyses with each of the relationship quality domains (communication, trust, intimacy, equality) as the dependent variable, and child marriage measures as the key independent variable. All analyses were performed in Stata 14. Data were weighted for complex survey design, including clustering of data.

### Integration of qualitative findings

Qualitative data collection was designed to generate information both on individual experiences and on the cultural milieu shaping both child marriage and its implications for women, with IDIs being used to capture the former and participatory FGDs the latter. The IDIs adopted a ‘life history’ approach to elicit biographical narratives focused on women’s own perceptions of the impact of child marriage and their lived experiences within marriage. Particular attention was paid to how being married at a young age had affected their life trajectories and their relationship with their husband. The FGDs captured normative perceptions of the impacts of child marriage from the perspectives of mothers and fathers with daughters aged 8–17. Focus group discussion participants were asked to reflect upon and map the life of a girl who marries early in their community (before age 18), compared to one who marries later.

Both the IDIs and FGDs were audio recorded, transcribed verbatim and then translated into English. An initial coding structure was developed based on the original research questions in addition to themes and topics that emerged during the interviews. The coding team of four investigators conducted inter-coder reliability tests on these transcripts by independently coding the first four out of 32 transcripts (12.5% of the total). For these transcripts, the team compared results and checked each other’s work to verify agreement in coding. Additions of new codes or changes in code definitions were determined via consensus among the research team and used to develop a final coding structure. Once all coding discrepancies were resolved, transcripts were coded using NVivo 10 qualitative data analysis software. Team members conducting the coding met frequently to resolve questions in coding and ensure consistency.

A qualitative inductive approach involving thematic assessment of the narratives was adopted to interpret the data. This approach allows research findings to emerge from the frequent, dominant or significant themes inherent in raw data. Emergent themes are then interpreted for meanings and messages through continuous investigation of narrative data for categories and linkages with the study objectives (Ryan and Bernard 2000). Resultant categories and emergent themes were analysed, discussed and harmonised by the research team and shared with study interviewers for validation. These themes emerged from informants’ narrations in response to questions related to their relationship with their husband in addition to how marriage had changed their lives.

## Results

### Quantitative findings

The relationship quality measures were only collected from currently married and recently divorced, separated or widowed women, which included 3971 women. As many as 12.4% of the sample did not know their age at first marriage. This missing information on the age at marriage variable, along with missing information on other variables, reduced the final analytical sample to 3396 women.

The average age of the women in the sample was 29.44 (SD:7.36) years, while 36% of the women had attended school, and 13.45% were employed (Table 1). The women were on average 7 years younger than their husbands, with 9% reported being in a polygynous relationship, and 30% of the women had experienced some form of intimate partner violence. Over 60% of the sample were married before reaching their eighteenth birthday, and almost 37% were married by or at age 15. The mean scores on the relationship quality domains ranged from an average of 69.68 (SD: 24.01) for trust scales to 60.46 (SD: 21.99) for the equality scale (Table 1). As shown in Table 2, there was a general negative relationship between relationship quality scores and age at first marriage, particularly among those married at age 15 or earlier.

**Table 1.** Descriptive statistics of study variables (n = 3396).

Variables	Distribution Mean (SD) or %
Communication	65.75 (26.41)
Trust	69.68 (24.01)
Intimacy	61.61 (21.16)
Equality	60.46 (21.99)
Married at 12 or before	6.41
Married at 13	3.76
Married at 14	8.59
Married at 15	17.75
Married at 16	12.64
Married at 17	12.87
Married at 18 or later	37.98
Current Age	29.44 (7.36)
Schooling	36.01
Employed	13.45
No of Children (n = 3900)	3.57 (2.13)
Intimate Partner Violence	29.71
Polygamy	8.63
Spousal Age Difference	7.06 (5.56)

**Table 2.** Mean (SD) of relationship quality scores by age at marriage among Ethiopian women aged 18–45 years (n = 3396).

Age at Marriage	12 Years or Earlier	13 Years	14 Years	15 Years	16 Years	17 Years	18 Years or Later
Relationship Quality Domains							
*Communication*	60.73 (24.38)	63.23 (30.12)	65.55 (24.18)	64.84 (25.37)	65.07 (25.36)	67.29 (25.38)	67.78 (25.83)
*Trust*	65.97 (23.54)	66.97 (24.84)	67.47 (22.43)	69.41 (23.98)	71.54 (24.36)	72.03 (24.27)	71.10 (23.19)
*Intimacy*	59.69 (19.81)	61.60 (20.73)	59.69 (19.90)	61.60 (20.73)	63.50 (19.73)	64.35 (20.85)	63.45 (21.46)
*Equality*	59.11 (20.39)	61.97 (22.64)	57.69 (20.58)	60.02 (21.26)	62.02 (20.82)	63.00 (21.28)	62.10 (22.45)

Results from the multivariate regression analysis (Table 3) provide support for this conclusion, though only at the very earliest ages of marriage, after accounting for intimate partner violence, spousal age difference, polygamous marriage and other factors. Marriage at or before age 12 reduced spousal communication by 4.79 points (β = −4.79, SE: 1.82), trust by 3.11 points (β = −3.11, SE: 1.54), intimacy by 3.61 points (β = −3.61, SE: 1.43) and equality by 2.57 points (β = −2.57, SE: 1.49) compared to marrying later (Table 3). Among other relationship factors, many often associated with child marriage, experiences of intimate partner violence and larger age differences between spouses, and polygamous marriages were significantly negatively associated with relationship quality (Table 3).

**Table 3.** Coefficients from multivariate linear regression analysis examining the association of child marriage (CM) with relationship quality (RQ) domains among Ethiopian women aged 18–45 years (n = 3396).

Variables	Communication^1^	Trust^1^	Intimacy^1^	Equality^1^
Married at 12 or Earlier	−4.79	−3.11	−3.61	−2.57
	(1.82)**	(1.54)**	(1.43)**	(1.49) *
Married at 13	−0.77	−0.88	0.40	1.99
	(2.26)	(2.04)	(1.43)	(1.59)
Married at 14	−0.40	−2.09	−1.88	−2.31
	(1.59)	(1.76)	(1.34)	(1.49)
Married at 15	−0.97	0.77	0.11	0.52
	(1.29)	(1.19)	(1.01)	(1.14)
Married at 16	−2.22	1.16	0.61	1.23
	(1.40)	(1.44)	(1.06)	(1.21)
Married at 17	−0.31	0.98	0.56	0.80
	(1.40)	(1.33)	(1.12)	(1.13)
Spousal Age Difference	−0.29	−0.14	−0.15	−0.15
	(0.09)***	(0.08) *	(0.07)**	(0.07)**
Polygamous Marriage	−11.15	−12.45	−8.86	−8.46
	(1.86)***	(1.90)***	(1.47)***	(1.57)***
Age in Years	−0.18	−0.23	−0.13	−0.20
	(0.08)***	(0.08)***	(0.06)**	(0.07)**
Schooling	1.56	−0.06	0.28	1.42
	(0.98)	(0.94)	(0.78)	(0.84) *
Currently Working	−1.33	−1.50	−3.10	−2.94
	(1.59)	(1.86)	(1.45)**	(1.56) *
No. of Children				
*1–2 Children*	4.74	1.61	0.93	1.20
	(1.55)**	(1.50)	(1.31)	(1.34)
*3–5 Children*	5.66	3.95	2.69	2.76
	(1.81)**	(1.54)**	(1.40) *	(1.47) *
*More than 5 Children*	8.48	6.68	3.60	4.39
	(2.16)***	(1.91)***	(1.79)***	(1.72)***
Intimate Partner Violence	−10.03	−10.24	−6.62	−7.91
	(1.24)***	(1.33)***	(1.14)**	(1.15)***
CM Community Level	0.11	−0.02	0.33	0.17
	(0.36)	(0.28)	(0.21)	(0.23)
RQ Community Level	0.69	0.51	0.63	0.71
	(0.06)***	(0.07)***	(0.05)**	(0.05)***
R-squared	0.21	0.17	0.24	0.23

Standard errors in parentheses

***p < 0.001,**p < 0.05, *p < 0.1

^1^Models also adjusted for respondent's religion, urban/rural residence & region and sample weights.

### Qualitative findings

The themes that emerged from the qualitative research indicated three main pathways by which child marriage diminished relationship quality. These factors included the lack of say in spousal selection and marriage timing, the non-readiness for sex at the time of marriage, and the reduced agency that women generally experienced within these marriages. While the first two factors were critical in the early stages of the marriage, the overall diminished agency led to decline in relationship quality over time. These factors made it hard for the women to experience sexual and emotional intimacy in their marriages, which was further exacerbated by their inability to express themselves or feel they were in control of their lives. Ultimately, these features of their marriage fuelled mistrust between spouses and increased violence within the relationship.

Reflecting on their lives, child brides repeatedly discussed how the lack of choice in selection of their spouse as well as the timing of marriage, key features that characterised many of their marriages, made their early marital experiences difficult and reduced the quality of their marriage. They particularly highlighted how these factors made it hard for them to experience physical and emotional intimacy with their partners. This in turn led to limited spousal communication, fostered mistrust between partners, especially regarding fidelity, and ultimately led to intimate partner violence. In the words of two child brides:

I didn’t have the energy and the capacity [to have sex]. Had I been like one of those people who get married being in love, I would have had the energy but at that time I was a child and knew nothing about it. So, I was so scared. When we tried to have sex, it would hurt me so we would fight about that … He would say to me, ‘It’s because you have someone else. It’s because he is waiting for you’ … As time went by, I got used to it. Because I was tired of the fights and I think he was going to other women.

Yes, it was through force [in reference to sex with her husband]. Whenever he wanted and according to his feelings. It was a while before I was able to be with him willingly and happily. The only reason I started being with him willingly now is because I started thinking about how if I keep resisting and saying no to him, he could go somewhere else for what he’s not getting from me and he could bring HIV/AIDS into our marriage and affect both my health and my children’s lives.

A related theme that came up in discussion emphasised child brides’ lack of readiness for sex because of their young age and inexperience, which made it difficult for them to enjoy their early sexual experiences. This, coupled with their fear of expressing their wishes or discussing their problems to find solutions, further aggravated the situation and led to the women either complying with their husband’s wishes regardless of their discomfort or exposing themselves to the risk of violence. In the words of two different women:

It used to be so painful for me when we had intercourse. But I couldn’t tell that to anyone. And when I refuse he used to beat me, splash water on me, put a rock on me and he waited till I get tired and took me afterwards.

*Interviewer:* Did you say no often?

*Respondent:* In the beginning, yes, because it was very uncomfortable until I got used to it.

*Interviewer:* Oh, so you used to say no in the beginning? What about afterwards? You stopped saying no once you got used to it?

*Respondent:* I just stayed quiet. We were living together so what could I say?

*Interviewer:* If you said I don’t feel like it today or I’m tired.

*Respondent:* That’s just starting a fight. He saw that as a sign of disrespect and starts an argument.

Finally, over the course of their relationship, the women shared how the general sense of powerlessness and lack of agency within their homes made it difficult to enjoy their marriage. The women were often unable to voice their opinions and take part in decision-making that pertained to themselves as well as their homes. This was particularly the case in the reproductive arena, where many women ultimately ended up with more children than they desired because of their inability to express their reproductive wishes. Many participants also described the difficulty in adapting to the need to ask for permission from their husbands in order to leave the house, to have visitors or make any type of decision. In the words of one woman:

*Interviewer:* That means the third child, right?

*Respondent:* The third child, then hmm … but between the two children there was a problem when I gave birth to my second child. She was so big in the in the stomach and it was a difficult pregnancy at the time and I delivered in Kuyera Hospital.

*Interviewer:* Where is Kuyera Hospital?

*Respondent:* It’s in Shashemene. They were planning to operate on me, but the child was pushed out by force and my womb was ruptured in the delivery. It was a very difficult moment for me and the doctor who helped me deliver the child told me that I should not give birth for seven or eight years, but because of my husband I got pregnant with my third child. Then, I became scared. How did I get pregnant when that doctor told me not to get pregnant?

Similarly, FGD participants also shared how early marriages are often unhappy because girls lack the physical and emotional maturity needed to handle marital responsibilities. Participants shared that child marriages are especially difficult in the beginning, particularly if the spouses were not acquainted before the wedding day, due to lack of familiarity with each other and with their new responsibilities. A mother (FGD participant) stated:

Before there was no law against it [meaning child marriage]. A girl who gets married early gets traumatised, won’t have a happy marriage, she will be physically abused. Nowadays, girls get married when they’re older, when they’re more physically ready and more mature and readier for a marriage.

## Discussion

Our quantitative data found significant negative association between very early marriage (at age 12 or lower) and relationship quality, and these findings were consistent across the different domains – trust, intimacy, communication and equality. However, the findings suggest that this is not broadly the case for child marriage, though, as discussed further below, the qualitative data suggest that women attribute many of their marital difficulties to having married early. Several mechanisms may be at play in shaping the observed relationship between early marriage and impaired relationship quality. Research suggests that early marriage often exposes women to elevated risk of intimate partner violence, reduced communication with their husbands/partners and limited ability to make decisions as compared to women who marry as adults, and there is evidence to suggest that this might be especially the case for those married very early (Kidman 2016; Wodon et al. 2017; Santhya, Haberland and Singh 2006; Godha, Hotchkiss and Gage 2013; McClendon et al. 2018).

The importance of these factors in shaping women’s marital experiences featured strongly in the qualitative analyses. The findings illustrate how lack of choice on when and whom to marry, non-readiness for sex in the early years of marriage, and general diminished agency, which often characterise the marriage of child brides, lead to dissatisfaction and compromised intimacy in marriage, ultimately discouraging spousal communication, increasing conflict and promoting IPV. The qualitative data suggest that there may be indirect effects between child marriage and relationship quality mediated by factors such as IPV, something we were unable to examine because of data limitations but which future studies should explore. However, clearly, our data suggest that, for the very young, child marriage has a direct negative association with relationship quality, and the findings remain robust even after adjusting for intimate partner violence and other factors that determine power distribution, such as spousal age difference and polygamous marriage, as well as community norms around marriage. While more research is needed to understand the mechanisms by which child marriage affects relationship quality, our study findings indicate that investing in programmes that provide couples with the skills and tools to develop healthy and positive relationships can go a long way in mitigating some of the negative effects of child marriage. In particular, interventions that provide couples with frameworks for negotiating conflict and improving basic levels of communication are needed, as these form the basis for a range of potential improvements in couple relations and other related outcomes, such as the utilisation of health services, de-escalation of conflict and women’s increased engagement in household decision-making processes.

### Limitations

This study has several limitations that need to be considered when interpreting the results. Both age at marriage and the relationship quality measures were self-reported and subject to recall and social desirability bias. Several procedures were adopted to minimise these errors, such as helping respondents remember their exact age at marriage by citing historical events and ensuring interviews were conducted in privacy. Finally, our qualitative data are limited and come from only two sites in Ethiopia, limiting generalisability to other locations. Despite these limitations, our study is, to our knowledge, the first to investigate the relationship between child marriage and relationship quality in Ethiopia (and one of few outside of the Western context) using large-scale population-based data, which was complemented by qualitative data. Moreover, the survey collected detailed measures on relationship quality and intimate partner violence, which are not routinely collected by national-level surveys. Finally, our study used exact ages at marriage to measure child marriage, which allowed for a more nuanced understanding of the gradient of risk at different ages at marriage.

## Conclusion

The study conducted a much more focused and in-depth assessment than has previously been possible of the ways in which child marriage influences the nature of the relationships that child brides have with their husbands, with significant ramifications for other areas of their lives. These findings provide support both to efforts to further justify policies and programmes aimed at ending child marriage and offer important insights for those working to mitigate its detrimental effects for those who were married as children. In particular, the findings point to the need to better understand the linkage between family formation patterns and relationship quality and the subsequent implications this may have for the lives of young women worldwide. Future research should seek to extend this further to better understand the implications of dissatisfaction within relationships on mental and physical health. Given these links and the centrality of marital relationships to the lives of many girls and young women globally, better understanding on how to improve the quality of these relationships should be a priority for future programme development.

## Appendix Appendix 1

**Table:** Factor loadings of items included in re-specified scales from the exploratory factor analysis of relationship quality scales (n = 3971)

Items	Loadings**
**Trust (Alpha = 0.91)**	
My partner is primarily interested in his own welfare	**
There are times when my partner cannot be trusted	**
My partner is perfectly honest and truthful with me	0.80
I feel I can trust my partner completely	0.85
My partner is truly sincere in his promises	0.88
I feel that my partner does not show me enough consideration	**
My partner treats me fairly and justly	0.78
I feel that my partner can be counted on to help me	0.76
**Intimacy (Alpha = 0.89)**	
I spend as much time with my partner as possible	0.81
I do as many activities with my partner as possible	0.83
I get so close to my partner that I am not sure When he or she begins and I end	0.72
My partner is a very important part of how I see myself	0.81
I think in terms of ‘we’ and ‘us’ instead of ‘I’ and ‘Me’	0.77
I can never get too close to my partner	**
**Equality (Alpha = 0.95)**	
My partner and I have equal power in the relationship	0.82
My partner shows as much affection to me as I think I show to him/her	0.82
My partner and I invest equal amounts of time and energy in the relationship	0.88
My partner and I are equally committed to working out problems that occur in our relationship	0.89
All things considered, my partner and I contribute an equal amount to the relationship	0.90
My partner and I deal with each other as equals	0.86
My partner treats me and respects me as an equal	0.83
My partner depends on me as much as I depend on him/her	0.69
**Communication (Alpha = 0.96)**	
*The Couple Discuss the Following:*	
Things that happened to him during the day	0.80
Things that happened to you during the day	0.86
Your worries or feelings	0.90
His worries or feelings	0.88
His hopes for the future	0.95
Your hopes for the future	0.90
The health needs of your children	**
What to spend money on	0.85
Things that happen in the community	0.89
The education of children	**
Birth spacing	**

**Loadings provided for final scale items only.
